# One-Step Multiplex PCR Reveals Selective Activation of Immunostimulatory Human Endogenous Retroviruses and Epigenetic Imbalance in Systemic Lupus Erythematosus

**DOI:** 10.3390/ijms27052474

**Published:** 2026-03-07

**Authors:** Ilaria Galliano, Pierluigi Sorgato, Cristina Calvi, Marzia Pavan, Anna Pau, Anna Massobrio, Roberto Albiani, Claudia Linari, Alice Geranzani, Anna Clemente, Paola Montanari, Stefano Gambarino, Francesco Licciardi, Massimiliano Bergallo

**Affiliations:** 1 Department of Public Health and Pediatric Sciences, University of Turin, Piazza Polonia 94, 10126 Turin, Italy; pierluigi.sorgato@edu.unito.it (P.S.); cristina.calvi@unito.it (C.C.); pauanna1@gmail.com (A.P.); anna.clemente@unito.it (A.C.); paola.montanari@unito.it (P.M.); stefano.gambarino@unito.it (S.G.); 2 Pediatric Laboratory, Department of Children’s Pathology and Care, Regina Margherita Children’s Hospital, Piazza Polonia 94, 10126 Turin, Italy; 3 Immunohematology and Transfusion Medicine, Regina Margherita Children’s Hospital, 10126 Turin, Italy; mapavan@cittadellasalute.to.it (M.P.); ralbiani@cittadellasalute.to.it (R.A.); 4 Laboratory Medicine, Regina Margherita Children’s Hospital, 10126 Turin, Italy; amassobrio@cittadellasalute.to.it (A.M.); clinari@cittadellasalute.to.it (C.L.); 5 Immunorheumatology Unit, Regina Margherita Children’s Hospital, University Hospital City of Health and Sciences of Turin, 10126 Turin, Italy; alice.geranzani@gmail.com (A.G.); francesco.licciardi@gmail.com (F.L.)

**Keywords:** one-step multiplex PCR, human endogenous retroviruses (HERVs), HERV-W, Syncytin-1, epigenetic dysregulation, systemic lupus erythematosus

## Abstract

Systemic lupus erythematosus (SLE) is characterized by chronic immune activation, enhanced type I interferon signaling, and epigenetic dysregulation, conditions that may promote the reactivation of human endogenous retroviruses (HERVs). Whether HERV activation in SLE is global or selective, however, remains unclear. We analyzed the expression of HERV-H, HERV-K, and HERV-W, along with the HERV-derived envelope genes Syncytin-1 and Syncytin-2, in samples from lupus patients and healthy controls. In parallel, we assessed the expression of the epigenetic repressors TRIM28 and SETDB1. HERV-H expression was comparable between groups, whereas HERV-K and HERV-W were significantly overexpressed in lupus patients. Syncytin-1 and HERV-W env transcripts were markedly increased in SLE, while Syncytin-2 expression was unchanged. Lupus patients showed reduced TRIM28 and increased SETDB1 expression, consistent with altered regulation of HERV repression pathways. Notably, HERV-H and HERV-W pol expression correlated with the type I interferon score, suggesting an association between interferon signaling and selective HERV activation. These findings indicate that SLE is associated with the selective activation of immunostimulatory HERV families, particularly HERV-W. The observed associations with interferon signaling suggest that HERV-W-related transcripts may represent disease-associated molecular signatures, warranting further mechanistic investigation.

## 1. Introduction

Human endogenous retroviruses (HERVs) are remnants of ancient exogenous retroviral infections that have become permanently integrated into the human germline and are transmitted vertically as part of the host genome. Collectively, HERV sequences account for approximately 8% of human genomic DNA, a proportion that greatly exceeds that of protein-coding genes [1,2]. Although the majority of HERV loci are defective due to accumulated mutations, deletions, or truncations, a substantial fraction retains transcriptional activity, and in some cases, intact open reading frames encoding functional proteins, particularly envelope (env) proteins [3,4,5].

Under physiological conditions, HERV expression is tightly controlled by multilayered epigenetic mechanisms, including DNA methylation, histone modifications, and chromatin remodeling. A central role in this repression is played by the KRAB zinc-finger protein (KRAB-ZFP) pathway, which recruits the scaffold protein TRIM28 (also known as KAP1) and promotes the deposition of repressive histone marks such as H3K9me3 through the histone methyltransferase SETDB1 [6,7,8]. This coordinated epigenetic machinery ensures the long-term silencing of retroelements, thereby preserving genome stability and preventing aberrant activation of innate immune responses triggered by retroviral nucleic acids or proteins [9]. While additional epigenetic regulators such as SUV39H1, DNMTs, HDACs, and MBD2 have been implicated in chromatin remodeling and SLE pathogenesis, the present study focused specifically on the TRIM28–SETDB1 axis due to its direct and well-characterized role in endogenous retroelement silencing.

Dysregulation of HERV expression has been increasingly implicated in chronic inflammatory and autoimmune diseases. Systemic lupus erythematosus (SLE) is a prototypical systemic autoimmune disorder characterized by a breakdown of immune tolerance, the production of pathogenic autoantibodies, immune complex deposition, and chronic activation of type I interferon (IFN-I) pathways [10,11,12]. Notably, IFN-I signaling, oxidative stress, and epigenetic instability, hallmarks of SLE, are all conditions that have been associated with the transcriptional reactivation of endogenous retroelements [13,14,15].

Persistent type I interferon signaling and epigenetic instability have been associated with altered chromatin accessibility and transcriptional reprogramming in autoimmune diseases [12,13,14,15]. Endogenous retroelements are particularly sensitive to such changes, as their silencing depends on DNA methylation and repressive histone modifications [6,7,8,9]. In inflammatory conditions, partial loss of these silencing mechanisms may facilitate selective retroelement reactivation [5].

Among the numerous HERV families, HERV-H, HERV-K (HML-2), and HERV-W have attracted particular attention due to their relatively recent evolutionary integration and the preservation of regulatory features. HERV-K elements are among the most transcriptionally active HERVs in humans and have been linked to cancer, neurodegeneration, and autoimmune diseases [16,17,18]. HERV-W is of particular interest because its env-derived proteins, including Syncytin-1, can activate innate immune receptors such as Toll-like receptor 4 (TLR4), inducing pro-inflammatory cytokine release and IFN-I production [19,20,21]. In contrast, HERV-H elements are strongly associated with pluripotency networks and embryonic stem cell identity, suggesting a regulatory landscape distinct from that of HERV-K and HERV-W in differentiated immune cells [22,23].

Despite mounting evidence supporting a role for HERVs in SLE, it remains unclear whether lupus-associated epigenetic alterations lead to a global derepression of endogenous retroviruses or instead selectively affect specific HERV families. Moreover, the contribution of host epigenetic repressors to this potential family-specific dysregulation has not been fully elucidated. In this study, we investigated the transcriptional activity of HERV-H, HERV-K, and HERV-W in peripheral blood samples from SLE patients and healthy controls. We further analyzed the expression of the HERV-W-derived envelope gene Syncytin-1 and the HERV-FRD-derived envelope gene Syncytin-2, included as a comparator env transcript, together with the key HERV repressors TRIM28 and SETDB1, to explore the mechanisms underlying selective HERV activation in SLE.

## 2. Results

### 2.1. Study Population

A total of 25 patients with a diagnosis of SLE were enrolled in the study. The control group consisted of 27 asymptomatic subjects of comparable age (*p* = 0.2186) and with a comparable sex distribution (*p* = 0.5623).

Detailed demographic and clinical characteristics of SLE patients and healthy controls are summarized in Table 1.

### 2.2. Differential Expression of pol Genes of HERV Families in Lupus

**Figure 1:**
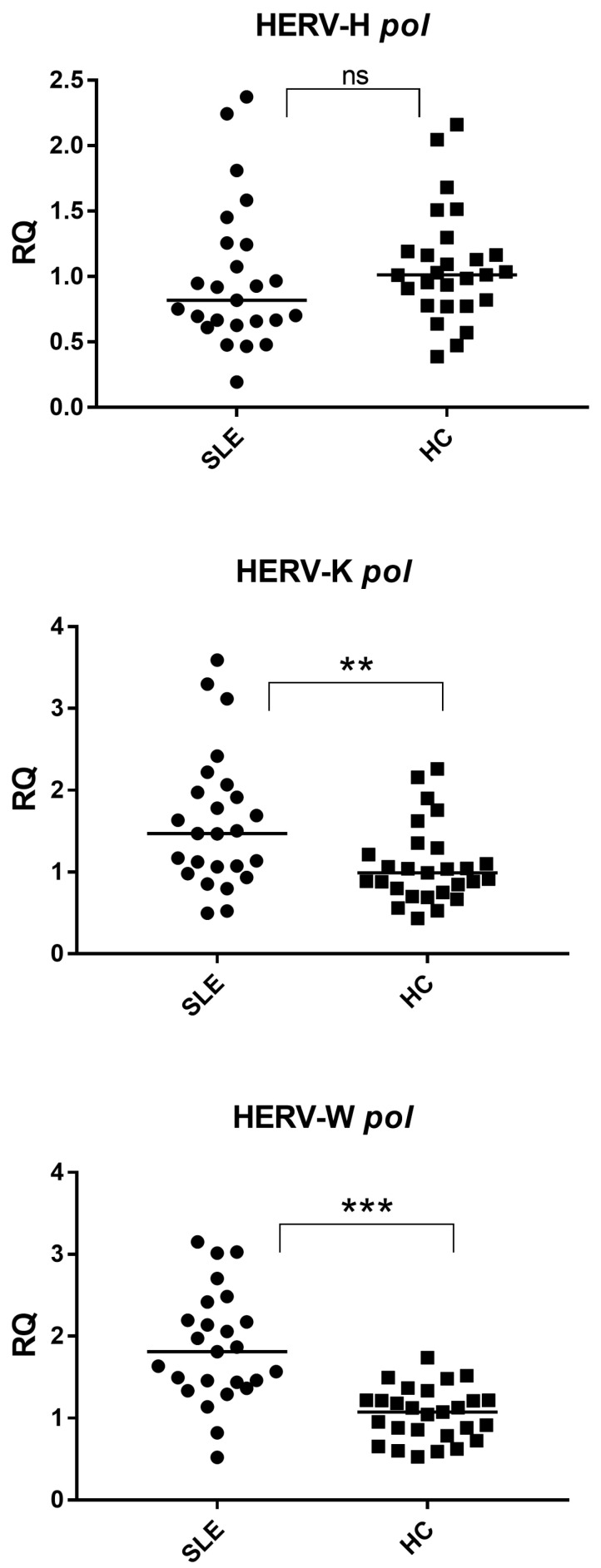
Expression of HERV-H, HERV-K, and HERV-W *pol* transcripts in healthy controls and lupus patients. HERV-K and HERV-W are significantly upregulated in lupus, whereas no significant difference was observed for HERV-H. SLE = Systemic lupus erythematosus; HC = Healthy control. RQ = Relative quantification; ΔΔCt method with GAPDH as the reference gene. Circles and squares indicate individual biological samples; horizontal lines represent median values. Statistical significance between groups was assessed using the Mann–Whitney test, with asterisks indicating *p*-values (** *p* < 0.01, *** *p* < 0.001, ns = not statistically significant).

Quantitative expression analyses revealed that HERV-H *pol* transcript levels did not significantly differ between the healthy controls and SLE patients (*p* = 0.1828) (Figure 1). In contrast, HERV-K pol and HERV-W *pol* transcripts were significantly overexpressed in samples derived from SLE patients compared with the controls (*p* = 0.0076 and *p* < 0.0001, respectively) (Figure 1).

These findings indicate that SLE is associated with a selective activation of specific HERV families, rather than a generalized derepression of endogenous retroviral elements.

HERV-H *pol*: SLE 0.82 (0.66–1.25), HC 1.01 (0.80–1.18); HERV-K *pol*: SLE 1.47 (1.06–1.97), HC 0.99 (0.78–1.26); HERV-W *pol*: SLE 1.81 (1.44–2.20), HC 1.07 (0.82–1.22).

### 2.3. Expression of Syncytins and HERV-W env

**Figure 2:**
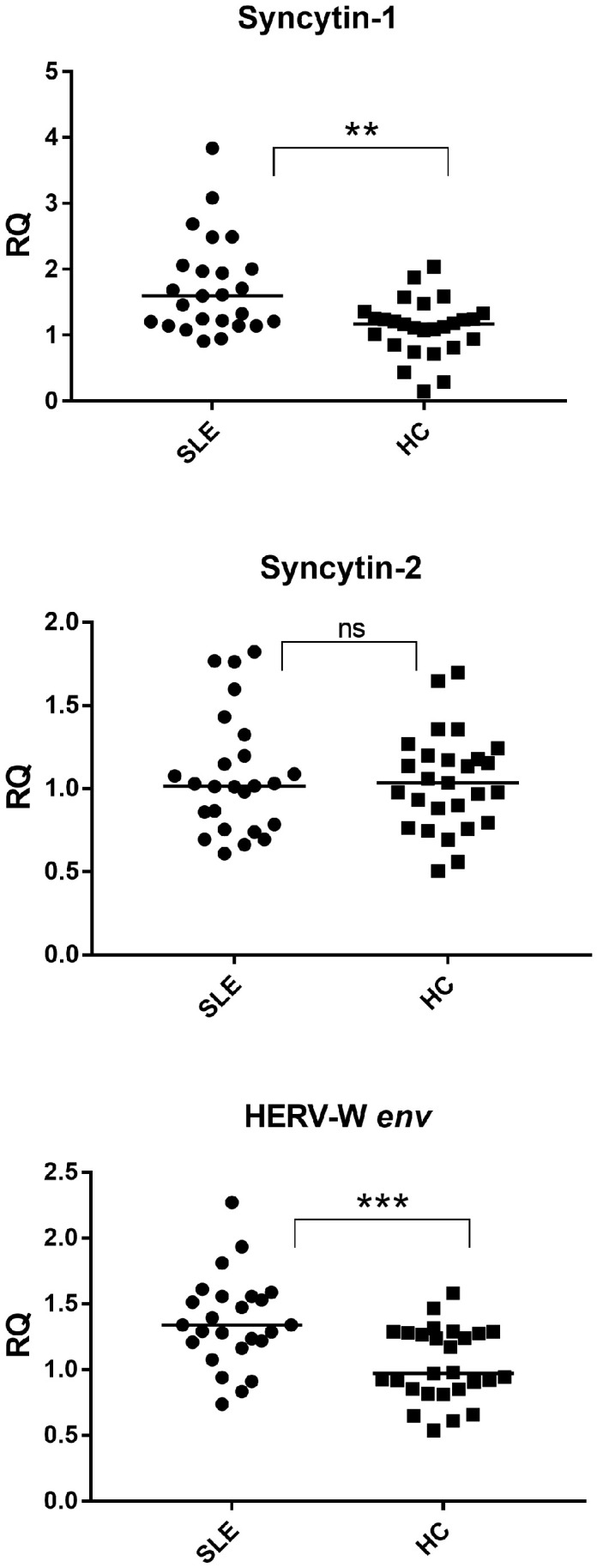
Expression of HERV-W-derived envelope genes Syncytin-1 and Syncytin-2, and HERV-W *env*, in healthy controls and lupus patients. Syncytin-1 and HERV-W *env* were significantly upregulated in lupus, whereas Syncytin-2 showed no significant difference between groups. SLE = Systemic lupus erythematosus; HC = Healthy control. RQ = Relative quantification; ΔΔCt method with GAPDH as the reference gene. Circles and squares indicate individual biological samples; horizontal lines represent median values. Statistical significance between groups was assessed using the Mann–Whitney test, with asterisks indicating *p*-values (** *p* < 0.01, *** *p* < 0.001, ns = not statistically significant).

Given the marked upregulation of HERV-W *pol* transcripts, we next examined the expression of HERV-W-derived envelope genes. Syncytin-1 expression was significantly increased in SLE patients compared with healthy controls (*p* = 0.0011), whereas Syncytin-2 expression did not differ significantly between the two groups (*p* = 0.9855) (Figure 2). The lack of Syncytin-2 upregulation supports the selective activation of HERV-W-related envelope transcripts.

Consistently, HERV-W *env* transcripts were also significantly upregulated in lupus samples (*p* = 0.0009) (Figure 2), closely mirroring the expression pattern observed for Syncytin-1.

Syncytin-1: SLE 1.60 (1.21–2.01), HC 1.17 (0.90–1.29); Syncytin-2: SLE 1.02 (0.78–1.20), HC 1.03 (0.84–1.19); HERV-W env: SLE 1.34 (1.21–1.55), HC 0.97 (0.85–1.28).

### 2.4. Expression of HERV Repressors TRIM28 and SETDB1

**Figure 3:**
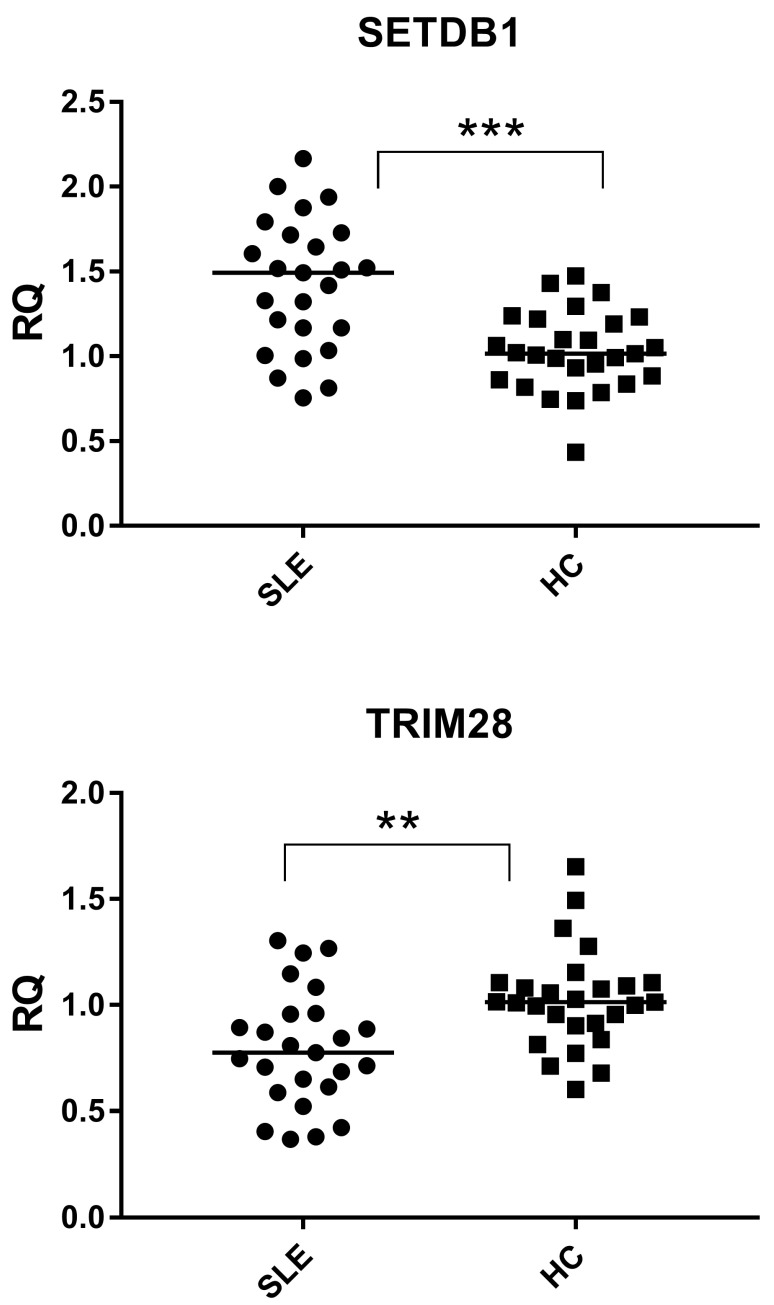
Expression of epigenetic repressors TRIM28 and SETDB1 in healthy controls and lupus patients. TRIM28 expression is reduced, whereas SETDB1 expression is increased in lupus samples. SLE = Systemic lupus erythematosus; HC = Healthy control. RQ = Relative quantification; ΔΔCt method with GAPDH as the reference gene. Circles and squares indicate individual biological samples; horizontal lines represent median values. Statistical significance between groups was assessed using Mann-Whitney test, with asterisks indicating *p*-values (** *p* < 0.01, ***p < 0.001).

To further characterize HERV-associated regulatory factors, we analyzed the expression of the epigenetic repressors TRIM28 and SETDB1. TRIM28 expression was significantly reduced in SLE patients compared with healthy controls (*p* = 0.0002), whereas SETDB1 expression was significantly increased (*p* = 0.0025) (Figure 3).

Thus, TRIM28 and SETDB1 displayed opposite expression trends in lupus, reflecting divergent transcript abundance of two key components of the HERV repression pathway.

SETDB1: SLE 1.49 (1.17–1.72), HC 1.02 (0.87–1.21); TRIM28: SLE 0.78 (0.61–0.96), HC 1.01 (0.91–1.10).

### 2.5. Correlation Between HERV Expression and Type I Interferon Score

**Figure 4:**
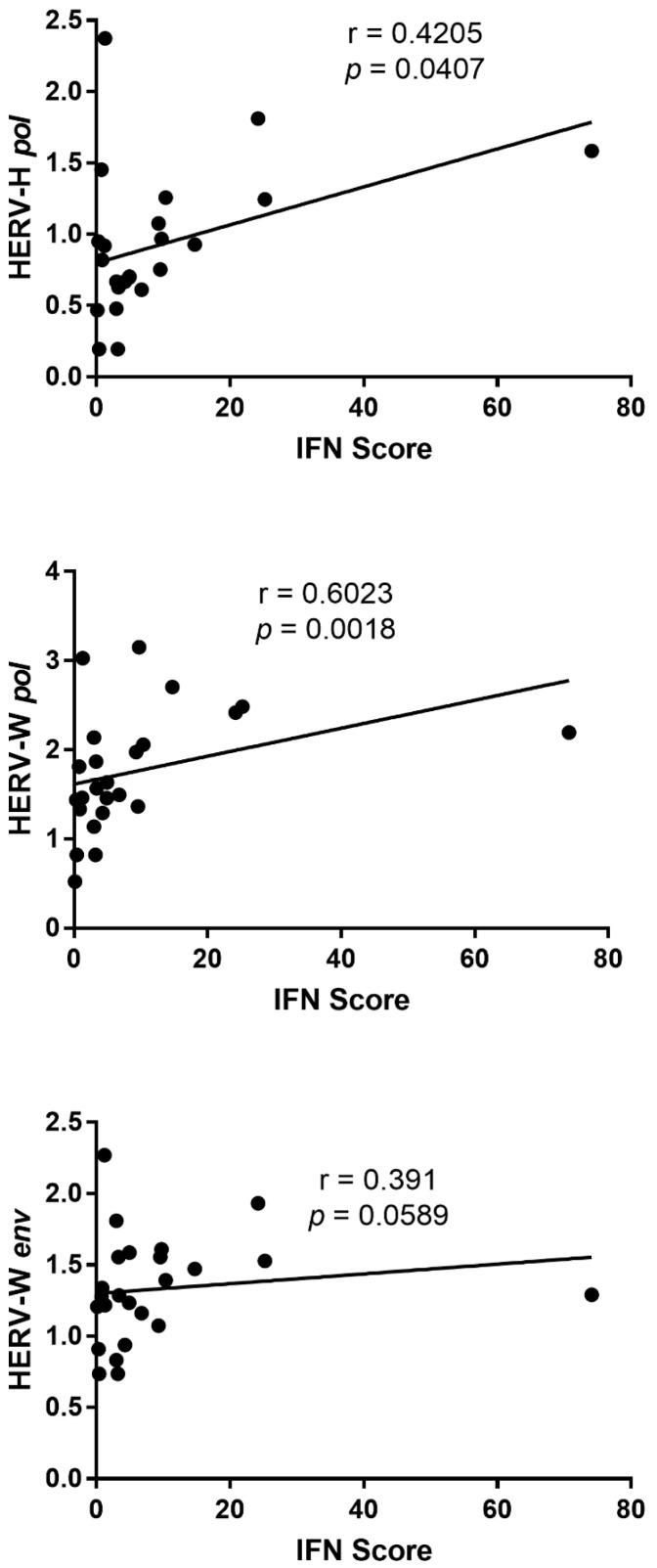
Correlation between type I interferon score and the expression of selected HERV transcripts in SLE patients. Scatter plots showing Spearman’s correlation between the type I interferon score and the expression levels of HERV-H *pol*, HERV-W *pol*, and HERV-W *env* transcripts in SLE patients. Circles indicate individual biological samples. Line: Linear regression line. Correlation coefficients (r) and *p* values are reported in each panel. No significant correlations were observed for the other analyzed targets.

Correlation analyses were performed to investigate the relationship between the expression levels of HERV transcripts and the type I interferon score in SLE patients. Correlation analyses were performed in all 25 SLE patients. The interferon score showed a significant positive correlation with the expression of HERV-H *pol* and HERV-W *pol* transcripts (Figure 4).

In contrast, no significant correlation was observed between the interferon score and HERV-W *env* expression, although a trend toward a positive association was detected (*p* = 0.0589).

No significant correlations were observed between the interferon score and the expression of other analyzed targets.

## 3. Discussion

In this study, we demonstrate that systemic lupus erythematosus is characterized by a selective dysregulation of endogenous retroviral elements, rather than by a global loss of HERV repression. While HERV-H expression remained unchanged, both HERV-K and HERV-W were significantly upregulated in lupus patients (Figure 1). These findings support the concept that distinct HERV families display differential sensitivity to the inflammatory and epigenetic milieu characteristic of SLE.

One plausible explanation for this selectivity lies in the intrinsic heterogeneity of HERV families with respect to genomic localization, regulatory sequences, and transcription factor responsiveness. HERV-K and HERV-W loci are frequently enriched in regulatory regions responsive to inflammatory transcription factors, including NF-κB and interferon-stimulated response elements [17,19,24]. Chronic type I interferon exposure, a defining feature of SLE, may therefore preferentially enhance transcription from these loci, either directly or indirectly through inflammation-induced chromatin remodeling [13,14]. In line with this model, we observed a significant positive correlation between the type I interferon score and the transcription of HERV-H and HERV-W *pol* genes (Figure 4). This finding supports an association between interferon-driven inflammatory activity and the increased transcription of selected endogenous retroviral families in SLE. Notably, the lack of a statistically significant correlation with HERV-W *env* expression suggests that interferon exposure may preferentially impact core retroviral transcription rather than envelope gene expression, pointing to an additional level of locus-specific regulation. In contrast, HERV-H elements are embedded in transcriptional networks linked to pluripotency and embryonic stem cell identity, suggesting that their regulation in differentiated immune cells relies on alternative, inflammation-insensitive mechanisms [22,23].

The epigenetic context provides an additional layer of selectivity. HERV-K and HERV-W elements rely heavily on the TRIM28-mediated recruitment of SETDB1 to establish and maintain H3K9 trimethylation-dependent heterochromatin [6,7,8]. Beyond its role in retroelement silencing, SETDB1 is also a key regulator of T-cell survival, differentiation, and immune tolerance, and its dysregulation has been directly implicated in autoimmune processes [25]. The reduced expression of TRIM28 observed in lupus patients (Figure 3) may be associated with the altered regulation of HERV repression pathways. However, the present data are limited to transcript-level measurements and do not provide direct evidence of epigenetic derepression at specific loci. Functional and chromatin-level analyses would be required to establish mechanistic links. In contrast, HERV-H loci may be more strongly controlled by DNA methylation or alternative histone modifiers, rendering them relatively resistant to fluctuations in TRIM28 expression [26].

The pronounced overexpression of HERV-W *env* and Syncytin-1 is of particular interest in the context of lupus-associated inflammatory activity (Figure 2). HERV-W-derived envelope proteins have been shown to act as potent immunostimulatory molecules capable of activating TLR4/CD14 signaling pathways, thereby promoting pro-inflammatory cytokine release and type I interferon production [19,20,21]. In SLE, such observations may be compatible with a model in which HERV-W expression and interferon signaling are biologically interconnected. However, the present transcript-level data do not establish functional directionality or causal relationships between these processes.

The differential expression of the two syncytins observed in our study provides additional biological context for the selective activation of HERV-W in lupus. Although both Syncytin-1 and Syncytin-2 originate from endogenous retroviral *env* genes, they derive from distinct HERV families (HERV-W and HERV-FRD, respectively) and are regulated by different promoter architectures and epigenetic landscapes [27]. Syncytin-1 expression is driven by regulatory regions that retain responsiveness to inflammatory transcription factors and interferon-stimulated pathways, making it particularly sensitive to the chronic inflammatory environment characteristic of SLE [19,21].

In contrast, Syncytin-2 is subject to tighter epigenetic control and exhibits a more restricted, tissue-specific expression pattern, primarily associated with placental development and immune tolerance at the maternal–fetal interface [27]. Its promoter region appears less responsive to inflammatory signaling and may rely more strongly on DNA methylation-dependent silencing mechanisms, which are not uniformly disrupted in lupus. This regulatory divergence may contribute to the absence of Syncytin-2 upregulation despite the robust induction of Syncytin-1 and HERV-W *env* transcripts.

The concomitant overexpression of HERV-W *env* is consistent with selective transcriptional activation within the HERV-W family. Individual HERV-W loci display variable sensitivity to epigenetic perturbations, and those retaining intact env open reading frames and accessible chromatin configurations may be more transcriptionally responsive in inflammatory contexts characterized by altered TRIM28 expression and interferon signaling. Together, these findings support the concept that endogenous retroviral activation in SLE may be both family- and locus-specific, with potential biological implications that warrant further mechanistic investigation. Importantly, the present study provides transcriptional evidence of selective HERV activation and associated regulatory signatures, but does not establish direct mechanistic causality.

**Figure 5:**
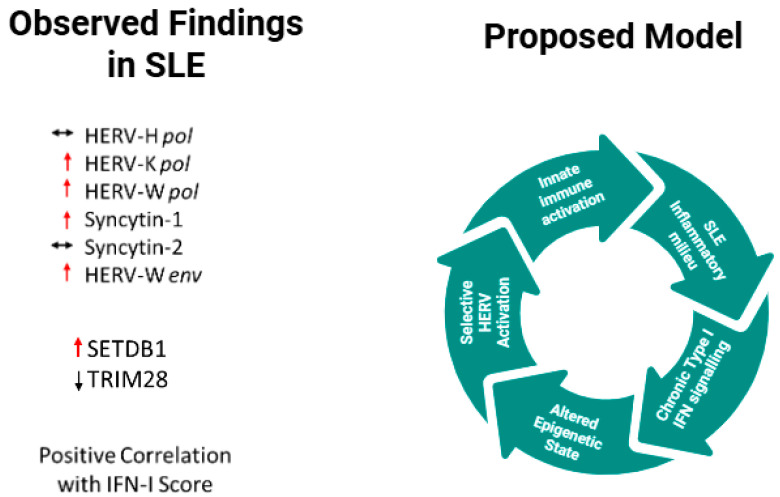
Proposed model of selective HERV activation in systemic lupus erythematosus. In the inflammatory milieu characteristic of SLE, chronic type I interferon signaling and alterations in epigenetic regulation (including reduced TRIM28 expression) may favor the selective activation of HERV-K and HERV-W families. Increased expression of HERV-W-derived envelope transcripts is hypothesized to be associated with innate immune activation. Green Arrows represent hypothetical relationships derived from transcript-level associations and do not imply experimentally demonstrated causality. Observed Findings in SLE: Red upward arrows indicate increased expression, black downward arrows indicate decreased expression, and the horizontal double arrows indicate no significant change.

A schematic model summarizing the observed transcript-level findings and the proposed mechanistic framework is presented in Figure 5.

This study has several limitations. First, the relatively limited sample size may reduce statistical power and limit the generalizability of the findings, warranting validation in larger independent cohorts. Although sex distribution was comparable between SLE patients and healthy controls, the limited number of male patients prevented adequately powered sex-stratified analyses. Therefore, potential sex-specific regulation of HERV transcription cannot be excluded and should be investigated in larger studies. Second, patients were sampled at heterogeneous time points during their disease course, and only a minority were evaluated at disease onset; therefore, HERV expression patterns may reflect combined effects of disease activity, duration, and ongoing treatment. Third, the cross-sectional design precludes causal or temporal inferences regarding the relationship between interferon signaling, epigenetic imbalance, and HERV activation. Fourth, treatment regimens were heterogeneous, and no multivariable adjustment was performed; therefore, treatment-related effects cannot be fully excluded, and the relative contribution of disease activity versus therapy cannot be definitively disentangled. Finally, although the assays were designed using degenerate primers targeting conserved regions to detect overall family-level transcription rather than locus-specific insertions, ancestry-related HERV insertion polymorphisms were not specifically assessed. Therefore, the generalizability of these findings to populations with different genetic backgrounds may be limited. Longitudinal studies in well-characterized and treatment-naïve cohorts will be essential to further clarify these associations. In addition, RNA was extracted from whole blood samples; therefore, the observed differences in HERV and epigenetic regulator expression may reflect not only transcriptional regulation but also variations in immune cell composition between SLE patients and healthy controls. Given that SLE is characterized by alterations in circulating leukocyte subsets, including changes in lymphoid and myeloid compartments, cell composition effects cannot be excluded. Cell type-specific analyses or computational deconvolution approaches will be required to disentangle transcriptional regulation from compositional variability.

Importantly, the present study was limited to transcript-level measurements in whole blood and was not designed to establish mechanistic causality. Accordingly, the biological interpretations proposed here should be regarded as hypothesis-generating and require functional validation.

Overall, our results strengthen the rationale for considering HERV-derived products, particularly HERV-W *env* and Syncytin-1, not only as markers of disease-associated epigenetic dysregulation, but also as candidate molecular signatures that may merit further investigation as potential biomarkers in future studies. In parallel, the altered expression of SETDB1 observed in lupus patients may have broader immunological consequences, given its established role in T-cell homeostasis and autoimmunity [25].

## 4. Materials and Methods

### 4.1. Patients and Study Design

The study included 25 patients affected by SLE and followed at the Immunorheumatology Unit of the Regina Margherita Children’s Hospital of Turin, Italy. All SLE patients had a definite diagnosis established by experienced pediatric rheumatologists and met internationally recognized classification criteria for SLE. Specifically, diagnosis was based on the fulfillment of the 2012 Systemic Lupus International Collaborating Clinics (SLICC) classification criteria [28], which require at least four criteria including at least one clinical and one immunologic criterion, or a biopsy-proven lupus nephritis in the presence of ANA or anti-dsDNA antibodies.

Patients with an uncertain or unconfirmed diagnosis were excluded. Disease activity at the time of sampling was assessed using the Systemic Lupus Erythematosus Disease Activity Index 2000 (SLEDAI-2K) [29].

Clinical data, including disease activity indices and laboratory parameters, were retrieved from medical records. The type I interferon score, assessed as part of routine clinical evaluation and obtained at the same time point as blood sampling, was included in correlation analyses with HERV expression data. The type I interferon score was calculated as the median of the expression of interferon-stimulated genes routinely assessed in the clinical laboratory, according to standardized procedures.

Blood samples were obtained from different SLE patients; however, not all samples were collected at the time of diagnosis. Only seven patients were sampled at disease onset, whereas the remaining patients were sampled at later time points after diagnosis and were receiving ongoing therapy at the time of blood collection.

The control group (HC) consisted of 27 asymptomatic subjects who were tested at the same hospital for routine laboratory examinations and whose results were all within the normal reference range. Subjects with any confirmed or suspected disease, including infections, cancer, autoimmune disorders, inflammatory diseases, neurological disturbances, or abnormal laboratory findings, were excluded from the study.

Peripheral blood samples were collected from SLE patients and from HC. Samples were stored in RNA-stabilizing solution as following: 200 μL of whole blood was added to 800 µL of RNApro solution (Biomole, Turin, Italy) in a 1.5-mL tube [30]; samples were vortexed and stored at −80 °C until extraction.

### 4.2. RNA Extraction

Total RNA was extracted from whole blood using the Maxwell automated extractor (Promega, Madison, WI, USA) with the RNA Blood Kit, which includes DNase treatment.

In each extraction session, a negative control was set up with sterile water as potential contamination control. RNA concentration and purity were assessed via UV spectrophotometry at 260/280 nm. NanoDrop (Thermo Fisher Scientific, Waltham, MA, USA) confirmed RNA concentrations within the acceptable range. RNA extracts were amplified without reverse transcription for GAPDH to rule out genomic DNA contamination. The absence of amplification confirmed that no significant genomic DNA contamination was present. RNA extracts were stored at −80 °C until use.

### 4.3. Transcription Levels of pol Genes of HERV-H, -K, and -W; env Genes of SYN1, SYN2, and HERV-W; and of TRIM28 and SETDB1 by Multiplex OneStep Real-Time PCR Assay

Transcription levels of the *pol* genes of HERV-H, HERV-K, and HERV-W, the *env* genes of SYN1, SYN2, and HERV-W as well as of TRIM28 and SETDB1 were quantified by multiplex OneStep real-time reverse transcription PCR (RT-qPCR) using probe-based (TaqMan) assays.

Primer and probe sets were selected from previously published and extensively validated methodological studies. In particular, assays targeting HERV *pol* transcripts were originally designed using degenerate primers amplifying conserved regions of the *pol* gene and have been shown to specifically detect HERV *pol* RNA in independent studies [31,32]. Assays targeting *env* genes of SYN1, SYN2, and HERV-W were derived from previously published protocols demonstrating reliable and differential detection of these transcripts [33,34]. Primer and probe sequences used in the present study are reported in Table 2. Probes labeled with fluorophores suitable for multiplex detection were employed, while primer and probe sequences remained unchanged.

Reverse transcription and amplification were performed in a single step using Reliance One-Step Multiplex Supermix (Promega, Madison, WI, USA) on an ABI 7500 Real-Time PCR System (Applied Biosystems, Foster City, CA, USA). The thermal cycling conditions were as follows: reverse transcription at 50 °C for 10 min, initial denaturation at 95 °C for 10 min, followed by 40 cycles of denaturation at 95 °C, and annealing/extension at 60 °C.

Three different multiplex reaction mixtures were used, as detailed in Table 2. Final concentrations of primers and probes were optimized for each target within the multiplex reactions and are reported in Table 3.

Gene expression levels were calculated as relative quantities (RQ) using the comparative ΔΔCt method [35]. Ct values of target genes were normalized to GAPDH, which was selected as the reference gene due to its stable expression in blood cells and its previous validation in our studies. GAPDH Ct values were compared between SLE patients and healthy controls, and no significant difference was observed (Mann–Whitney U test, *p* = 0.9710), supporting its stability as a reference gene in this cohort. Relative expression levels were calculated with respect to healthy controls.

Each run included a no-template control (NTC) to exclude contamination or non-specific amplification. The specificity of the assays and the representativeness of the amplified products for HERV transcripts were supported by prior methodological validations and by the use of conserved target regions combined with probe-based chemistry.

### 4.4. Statistical Analysis

Statistical analyses were performed using GraphPad Prism software (version 7.0). Data distribution was assessed using the Shapiro–Wilk test and showed a non-normal distribution of the analyzed variables. Therefore, comparisons between independent groups were conducted using non-parametric tests, specifically the Mann–Whitney U test. Correlations were conducted using Spearman’s correlation test. Categorical variables, including sex distribution, were analyzed using Fisher’s exact test. A *p*-value < 0.05 was considered statistically significant.

## Figures and Tables

**Table 1:** Demographic and clinical characteristics of SLE patients and HC.

Characteristic	SLE (*n* = 25)	HC (*n* = 27)
Sex (*n*, %)		
Male	7 (28)	10 (37)
Female	18 (72)	17 (63)
Age: median, IQR (25–75%) (years)	17.2, 14.8–19.6	13.5, 11.6–14.5
At diagnosis, *n* (%)	7 (28)	
Disease duration at sampling: median, IQR (25–75%) (months)	30.6, 3.8–70.1	
SLEDAI-2K score at sampling: median, IQR (25–75%)	2, 0–6	
Anti–dsDNA levels, IU/mL: median, IQR (25–75%)	18, 4–89	
Ongoing Treatment (*n*, %)		
Azathioprine	2 (8)	
Hydroxychloroquine	18 (72)	
Oral Prednisone	12 (48)	
Mycophenolate Mofetil	4 (16)	
Anakinra	1 (4)	
Intravenous Methylprednisolone	1 (4)	
Belimumab	1 (4)	
Calcineurin inhibitors (tacrolimus or cyclosporine)	3 (12)	

SLE = Systemic lupus erythematosus; HC = Healthy control; *n* = number; IQR = Interquartile range. SLEDAI-2K = Systemic Lupus Erythematosus Disease Activity Index 2000. Anti-dsDNA = Anti-double-stranded DNA antibodies. Other immunomodulatory agents (e.g., anakinra, eculizumab) were used in selected patients for specific clinical indications and are not reported in the table.

**Table 2:** Primer and probe sequences used for multiplex RT-qPCR assays.

Name	Primer/ Probe	Sequence
HERV-H *pol*	Forward	5′-TGGACTGTGCTGCCGCAA-3′
	Reverse	5′-GAAGSTCATCAATATATTGAATAAGGTGAGA-3′
	Probe	6FAM-5′-TTCAGGGACAGCCCTCGTTACTTCAGCCAAGCTC-3′-BHQ1
HERV-K *pol*	Forward	5′-CCACTGTAGAGCCTCCTAAACCC-3′
	Reverse	5′-TTGGTAGCGGCCACTGATTT-3′
	Probe	CY5-5′-CCCACACCGGTTTTTCTGTTTTCCAAGTTAA-3′–BHQ2
HERV-W *pol*	Forward	5′-ACMTGGAYKRTYTTRCCCCAA-3′
	Reverse	5′-GTAAATCATCCACMTAYYGAAGGAYMA-3′
	Probe	JOE-5′-TYAGGGATAGCCCYCATCTRTTTGGYCAGGCA-3′-BHQ1
Syncytin-1 *env*	Forward	5′-ACTTTGTCTCTTCCAGAATCG-3′
	Reverse	5′-GCGGTAGATCTTAGTCTTGG-3′
	Probe	6FAM-5′-AAACTACAAATGGAGCCCAAGATGC-3′-BHQ1
Syncytin-2 *env*	Forward	5′-GCCTGCAAATAGTCTTCTTT-3′
	Reverse	5′-ATAGGGGCTATTCCCATTAG-3′
	Probe	CY5-5′-TGATATCCGCCAGAAACCTCCC-3′-BHQ2
HERV-W *env*	Forward	5′-CTTCCAGAATTGAAGCTGTAAAGC-3′
	Reverse	5′-GGGTTGTGCAGTTGAGATTTCC-3′
	Probe	JOE-5′-TTCTTCAAATGGAGCCCCAGATGCAG-3′-BHQ1
SETDB1	Forward	5′-GCCGTGACTTCATAGAGGAGTATGT-3′
	Reverse	5′-GCTGGCCACTCTTGAGCAGTA-3′
	Probe	6FAM-5′-TGCCTACCCCAACCGCCCCAT-3′-BHQ1
TRIM28	Forward	5′-GCCTCTGTGTGAGACCTGTGTAGA-3′
	Reverse	5′-CCAGTAGAGCGCACAGTATGGT-3′
	Probe	JOE-5′-CGCACCAGCGGGTGAAGTACACC-3′-BHQ1
GAPDH	Forward	5′-ATGCTGGCGCTGAGTACGT-3′
	Reverse	5′-AGCCCCAGCCTTCTCCAT-3′
	Probe	CY5-5′-TGGAGTCCACTGGCGTCTTCACCA-3′-BHQ2

**Table 3:** Composition of multiplex RT-qPCR reaction mixtures.

Multiplex Mix	Target	Forward Primer (nM)	Reverse Primer (nM)	Probe (nM)
MIX HERV-*pol*	HERV-H (*pol*)	300	300	150
	HERV-K (*pol*)	400	200	200
	HERV-W (*pol*)	900	900	250
MIX GAPDH–SETDB1–TRIM28	GAPDH	300	300	150
	SETDB1	300	300	150
	TRIM28	300	300	150
MIX HERV-*env*	SYN1 (*env*)	300	300	150
	SYN2 (*env*)	300	300	150
	HERV-W (*env*)	300	300	150

All reactions were performed using Reliance One-Step Multiplex Supermix (Promega). Final primer and probe concentrations are reported. nM = nanoMolar.

## Data Availability

The results of this article will be shared at the aggregate/population level upon reasonable request to the corresponding author.
